# Mobile health technologies supporting colonoscopy preparation: A systematic review and meta-analysis of randomized controlled trials

**DOI:** 10.1371/journal.pone.0248679

**Published:** 2021-03-18

**Authors:** Maria El Bizri, Mariam El Sheikh, Ga Eun Lee, Maida J. Sewitch

**Affiliations:** 1 Centre for Outcomes Research & Evaluation, Division of Clinical Epidemiology, Research Institute of the McGill University Health Centre, Montreal, Quebec, Canada; 2 Department of Medicine, Division of Gastroenterology, McGill University, Montréal, Québec, Canada; Witten/Herdecke University, GERMANY

## Abstract

**Background:**

Mobile health (mHealth) technologies are innovative solutions for delivering instructions to patients preparing for colonoscopy.

**Objective:**

To systematically review the literature evaluating the effectiveness of mHealth technologies supporting colonoscopy preparation on patient and clinical outcomes.

**Methods:**

MEDLINE, EMBASE, CINAHL and CENTRAL were searched for randomized controlled trials (RCTs) that evaluated the effectiveness of mHealth technologies for colonoscopy preparation on patient and clinical outcomes. Two reviewers independently assessed study eligibility, extracted data, and appraised methodological quality using the Cochrane Risk-of-Bias tool. Data were pooled using random effects models and when heterogeneity, assessed using I^2^, was statistically significant, a qualitative synthesis of the data was performed. Publication bias was assessed using a funnel plot.

**Results:**

Ten RCTs (3,383 participants) met inclusion criteria. MHealth interventions included smartphone apps, SMS text messages, videos, camera apps, and a social media app. Outcomes were bowel cleanliness quality, user satisfaction, colonoscopy quality indicators (cecal intubation time, withdrawal time, adenoma detection rate), adherence to diet, and cancellation/no-show rates. MHealth interventions were associated with better bowel cleanliness scores on the Boston Bowel Preparation Scale [standardized mean difference (SMD) 0.57, 95%CI 0.37–0.77, I^2^ = 60%, p = 0.08] and the Ottawa Bowel Preparation Scale [SMD -0.39, 95%CI -0.59–0.19, I^2^ = 45%, p = 0.16], but they were not associated with rates of willingness to repeat the colonoscopy using the same regimen [odds ratio (OR) 1.88, 95%CI 0.85–4.15, I^2^ = 48%, p = 0.12] or cancellations/no-shows [OR 0.96, 95%CI 0.68–1.35, I^2^ = 0%]. Most studies showed that adequate bowel preparation, user satisfaction and adherence to diet were better in the intervention groups compared to the control groups, while inconsistent findings were observed for the colonoscopy quality indicators. All trials were at high risk of bias for lack of participant blinding. Visual inspection of a funnel plot revealed publication bias.

**Conclusions:**

MHealth technologies show promise as a way to improve bowel cleanliness, but trials to date were of low methodological quality. High-quality research is required to understand the effectiveness of mHealth technologies on colonoscopy outcomes.

## Introduction

Worldwide, colorectal cancer is the second and third most commonly diagnosed cancer in males and females, respectively [1]. Colorectal cancer screening has been recommended by the U.S. Preventive Services Task Force since 1996 [2], and by the Canadian Task Force on Preventive Health Care since 2001 [3]. Effective colorectal cancer screening depends, in part, on colonoscopy, an invasive procedure that permits visualization of the colon, performance of biopsies, and removal of abnormal lesions. Undergoing timely colonoscopy may reduce by half the number of colorectal cancer deaths following an abnormal result to the initial stool-based screening test [4]. However, up to 25% of patients undergoing colonoscopy do not achieve adequate bowel cleanliness [5], which can result in poor visualization of the colon, missed pathology, and procedural difficulties and complications. Not only does inadequate bowel cleanliness waste resources in terms of capacity, time, and money, it also exposes patients to additional risks associated with undergoing repeat colonoscopy [6–11].

Bowel preparation for patients involves multiple and complex steps. These steps include restriction of diet, fluids and medications in up to 7 days before the colonoscopy, and consumption of a laxative in the 24 hours prior to the colonoscopy. Adhering to the bowel preparation instructions is essential as nonadherence to the laxative and dietary instructions is associated with a nearly 5-fold increased risk of inadequately cleansed bowels [12]. The quality of the bowel preparation is assessed with various scales, of which the Boston Bowel Preparation Scale (BBPS) and the Ottawa Bowel Preparation Scale (OBPS) are two of the most commonly used. However, the two scales are not directly comparable as they assess different criteria and the better scores go in opposite directions. Total BBPS is obtained by summing scores for each segment of the bowel, and ranges from 0 (very poor) to 9 (excellent). A total BBPS of ≥ 6 with scores of ≥ 2 per segment is considered the optimal threshold for adequate bowel preparation. In contrast, total OBPS is obtained by summing scores for each bowel segment, which ranges from 0 (mucosa clearly visible) to 4 (solids impedes vision), plus the total colon fluid score, which ranges from 0 (small amount of fluid) to 2 (large amount of fluid) [13]. The total OBPS ranges from 0 (excellent) to 14 (inadequate), and there is no threshold for adequacy. These scale differences make comparisons of bowel preparation quality across studies difficult.

Given the complexity of preparing the bowel for colonoscopy, education is required for patients to satisfactorily perform the bowel preparation. Educational tools including booklets, cartoons and text-messaging have been developed to improve the quality of bowel preparation [14]. A systematic review showed that patient educational interventions are associated with improved bowel cleanliness quality compared to usual care (verbal or written instructions) [15]. More recently, mobile health (mHealth) technologies have been developed to educate patients on bowel preparation for colonoscopy. MHealth technologies are innovative tools shown to improve access to evidence-based care and better inform and actively engage patients in their own care [16]. A 2019 meta-analysis of six randomized controlled trials (RCTs) and observational studies of smartphone apps to support patient preparation for colonoscopy reported a summary odds ratio (OR) of 2.67 (95% confidence interval (CI) = 1.00–7.13) for adequate bowel preparation and an overall mean difference in the BBPS of 0.90 (95%CI = 0.50–1.30) [17]. These findings suggest smartphone apps are effective at improving bowel cleanliness compared to usual care [17]. However, not all mHealth interventions designed to educate patients preparing for their colonoscopy appointments were included in the systematic review.

MHealth technologies offer innovative and wide-reaching solutions to deliver rigorous bowel preparation instructions in a portable, timely, easily accessible and potentially low-cost manner. Thus, the purpose of the present systematic review was to summarize and critically evaluate the available evidence on mHealth technologies that support patients preparing for their colonoscopy appointments.

## Methods

The protocol for this systematic review was registered in PROSPERO on November 4, 2019. The major discrepancy between the protocol and the study methodology employed is that the trial outcomes have been expanded to include adherence to diet and cancellation/no-show rates. Cancellations and no-shows were combined given they are a heterogeneous collection of cancelled, rescheduled and missed appointments [18]. We followed the PRISMA Checklist in preparing our paper for publication. Research ethics board approval was not required for this study that used published aggregate data.

### Data sources and searches

On May 4^th^, 2018, one co-author (GEL) and a medical librarian performed a systematic search of four databases including MEDLINE Daily and E-Pub Ahead of Print, In-Process, Other Non-Indexed Citations (Ovid), EMBASE Classic + Current (Ovid), CINAHL Plus with Full Text (EBSCOhost), and the Cochrane Central Register of Controlled Trials (CENTRAL) for observational studies and randomized controlled trials that compared colonoscopy outcomes in patients given smartphone-based technologies with patients given usual-care. The MEDLINE search strategy served as the reference search strategy and efforts were made to replicate this strategy across all databases (S1 Table). Searches were restricted to studies published between 1996 and 2017. We also searched clinicalTrials.gov for ongoing trials. On November 28, 2019, we updated our search (2018–2019) (S2 Table). Searches included subject headings (Medical Subject Heading in MEDLINE, EMTREE terms in EMBASE, subject terms in CINAHL, and subject headings in CENTRAL and keyword variations of three major concepts in the primary research question: (colonoscopy OR bowel preparation) AND mobile phones. Additional relevant articles were identified from reference lists of included full-text studies. Cross-sectional studies, qualitative studies, case reports or case series, theses, literature reviews, commentaries, editorials, and conference abstracts were excluded, as were studies without comparison groups or those that used historical controls.

Records found after systematic searching were pooled and deduplicated using the “Find Duplicates” function in Endnote reference management software. The default deduplication function in EndNote did not remove all duplicates; thus, records were further deduplicated using an adapted version of the Bramer et al method of deduplication [19, 20]. Studies were then uploaded to Rayyan, a free web and mobile app, for initial screening of abstracts and titles followed by full-text review [21].

### Study selection

Two reviewers independently applied inclusion and exclusion criteria to all studies. Eligible studies enrolled adults aged 18 and over who were scheduled for outpatient colonoscopy and owned or had access to mobile devices. Included settings were endoscopy units and gastroenterology or other outpatient clinics where colonoscopy is performed. Inpatient colonoscopies were excluded.

**Fig 1:**
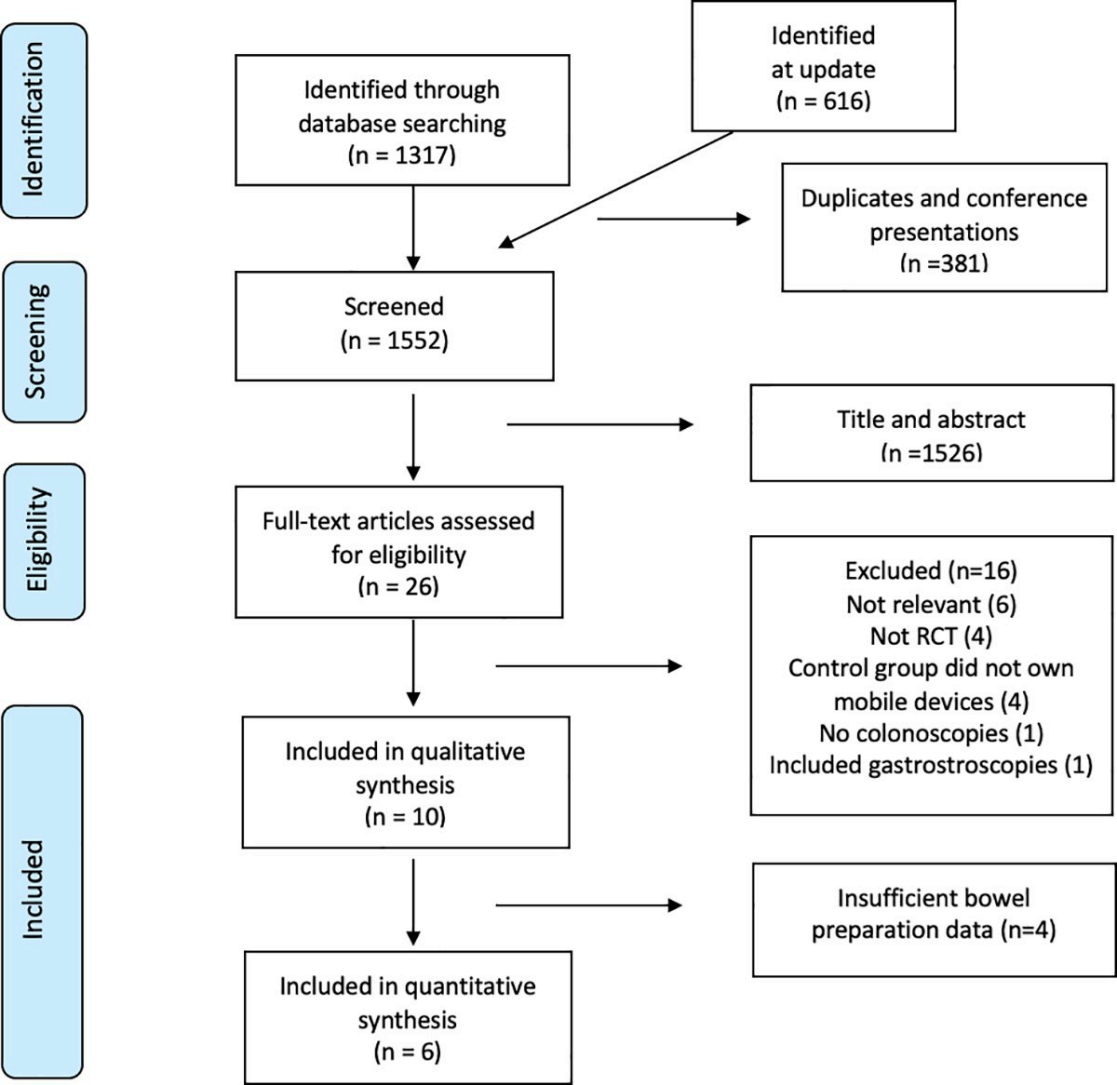
PRISMA flow diagram of article search results and selection.

Publications deemed potentially relevant by either reviewer during the title and abstract screening stage were carried to full-text review, and disagreements were resolved by consensus. The number of studies included at each stage and reasons for exclusion during full-text screening were recorded using the Preferred Reporting Items for Systematic Reviews and Meta-Analyses (PRISMA) flow diagram [22] (Fig 1). The full-text screening was restricted to studies published in English.

### Data extraction and quality assessment

Two reviewers independently extracted data using a template created with Microsoft Excel. A pilot test was run with three studies, and template adjustments were made accordingly. Data extracted included study characteristics (authors, year of publication, country, clinical setting), patient characteristics (sample size, age, sex, inclusion and exclusion criteria, indication for colonoscopy), and intervention characteristics (type of mobile technology, contents of treatment and control interventions). Data on clinical (bowel cleanliness quality, adequate bowel preparation, cecal intubation time, withdrawal time, adenoma detection rate, cancellation/no-show rate) and patient outcomes (user satisfaction, adherence to diet) were extracted where applicable.

Reviewers, independently and in duplicate, assessed the risk of bias of included studies using the Cochrane Collaboration’s risk of bias instrument for randomized trials. Each trial was evaluated for random sequence generation, allocation concealment, selective reporting, blinding of participants and personnel, blinding of outcome assessment, and incomplete outcome data. Each item was scored as high, low or unclear risk of bias. Disagreements were resolved by discussion or, if necessary, third-party adjudication.

### Data synthesis and analysis

Random-effects meta-analyses were conducted to determine the relationship between mHealth interventions and the various outcomes using the method proposed by DerSimonian and Laird [23]. Forest plots were presented according to outcome (standardized mean differences (SMD) and ORs); only one mHealth technology per study was included. The *I*^2^ statistic was calculated to assess the percent of variation across studies that is due to heterogeneity rather than chance [24, 25]. When the *I*^2^ statistic was statistically significant, data from these studies were not pooled, and a qualitative synthesis was performed instead. Sensitivity analysis on adequate bowel preparation was performed for the studies conducted in South Korea and the studies with fewer high-risk of bias domains. Publication bias was assessed for the outcome adequate bowel preparation via visual inspection of a funnel plot. All analyses were performed in R using the “metafor” and “meta” packages (version 3.3.0).

### Role of the funding sources

This study was conducted with financial support from the Canadian Institutes for Health Research (PIC392487), the Department of Medicine, McGill University, and the Research Institute of the McGill University Health Centre. The funding sources had no role in the design, execution, analyses or interpretation of the data.

## Results

The initial plan was to include both observational studies and RCTs in this systematic review. However, of the three observational studies identified, two were small feasibility studies that utilized historical database controls [26, 27] in which mobile phone ownership was unknown. Thus, with only one observational study meeting inclusion criteria [28], the decision was taken to restrict the systematic review to RCTs.

The initial electronic search of four databases identified a total of 1317 potentially relevant publications (S1 Table). After excluding duplicates and screening of titles and abstracts, 18 studies were eligible for full-text review. Of these, 7 full-text articles met the study criteria. The results of the update identified 616 potentially relevant publications (S2 Table). After excluding duplicates and conference presentations, and screening titles and abstracts, 4 studies were taken to full-text review. Of these, 3 studies met our study criteria (Fig 1).

### Description of included studies

Ten RCTs (3,383 participants) met the study eligibility criteria [29–38]. Characteristics of the included RCTs are summarized in Table 1. All studies were published between 2014 and 2019. Half (50%) the studies were conducted in South Korea [29–31, 33, 35], 2 in China [32, 38], and one each in Lebanon [36], Spain [34], and Germany [37]. Studies took place in university hospitals [31, 35, 36, 38], health examination clinics [30], tertiary-care hospitals [33, 37], and hospital outpatient colonoscopy clinics [29, 32, 34]. Participants ranged in age from 18 to 80 years, with average age ranging from 44.4 to 57.6 years. Only 3 studies explicitly stated smartphone ownership as an inclusion criterion [29, 34, 36]. Wang et al [38] evaluated two mHealth technologies (SMS, WeChat) compared to usual care while other studies evaluated only one.

**Table 1:** Characteristics of the trials on m-Health technologies to support outpatient colonoscopy preparation (N = 10).

Author, Year	Country	Setting	Sample size	Age mean±SD (range)	Sex	Inclusion criteria	Indication for colonoscopy
					n (% male)		
**Sharara et al., 2017 [36]**	Lebanon	University hospital private clinics, elective colonoscopy	160; 80 smartphone app 80 control	Overall 53.8±12.9	App	Age 18 and older, smartphone ownership	Screening
				(range 20–79)	52 (65.0) Control		Surveillance
					37 (46.3)		
**Lee et al., 2015 [33]**	South Korea	Tertiary hospital, outpatient colonoscopy	390; 126 SMS 126 Telephone 137 Control	SMS 45.7±12.4 Telephone 46.0 ±12.2 Control 47.1±11.8	SMS	Age over 18, screening colonoscopy	Screening
					76 (59.8) Telephone		
					79 (62.7) Control		
					73 (53.3)		
**Back et al., 2017 [29]**	South Korea	Academic referral centre, elective colonoscopy outpatient clinic	320; 160 AV 160 Control	AV 55.4±12.8	AV	Age 20–80, smartphone ownership	Screening Diagnostic
				Control 57.6±13.1	77 (55.4) Control		
					81 (56.3)		
**Lorenzo-Zuniga et al., 2015 [34]**	Spain	Outpatient	260; 108 App 152 Control	App 52.5±14.0	App	Age 18 and older, smartphone ownership	Screening Surveillance Diagnostic Afternoon colonoscopy
		elective colonoscopy		Control 48.3±13.5 (range 21–75)	48 (44.4) Control		
					60 (39.5)		
**Kang et al., 2016 [32]**	China	Hospital outpatient colonoscopy	770; 387 WeChat 383 Control	WeChat 44.4±13.2	WeChat	Age 18–80, access to WeChat themselves or through family member in same household	Screening Surveillance Diagnostic
				Control 45.5±13.0	202 (52.1) Control 191 (49.8)		
**Jung et al., 2017 [31]**	South Korea	University hospital elective outpatient colonoscopy	43; 19 App 24 No-app	App	App	Age 19–65, scheduled for elective colonoscopy	Screening Surveillance Diagnostic
				47.4±8.1 Control 51.0±7.6	13 (68) No-app		
				Total	11 (46)		
				49.4±7.9	Total		
					24 (56)		
**Park et al., 2014 [35]**	South Korea	University hospital, elective outpatient colonoscopy	271; 136 SMS 135 No-SMS	SMS 53.7±10.4	SMS	Age 18–80, scheduled for colonoscopy	Screening Surveillance Diagnostic
				No-SMS 55.8±12.3 Total 54.7±11.4	63 (46.3) No-SMS		
				(range 20–80)	63 (46.7) Total		
					126 (46.5)		
**Wang et al., 2019 [38]**	China	University medical centre open access endoscopy unit	393; 127 WeChat 128 SMS 125 Control	WeChat	WeChat	Age 18–80, routine diagnostic outpatient colonoscopy, access to WeChat or SMS themselves or close family member	Diagnostic
				48.9±13.0	79 (61.7) SMS		
				SMS	70 (54.3) Control		
				52.6±12.7	68 (53.5)		
				Control			
				51.5±12.1			
**Jeon et al., 2019 [30]**	South Korea	Health examination clinic	281; 140 AV 141 Control	AV 46.7±9.9 Control 49.9±9.6 Total	AV	Age >30, screening colonoscopy	Screening
				48.3±9.9	80 (57.1) Control		
					81 (57.4)		
**Walter et al., 2019 [37]**	Germany	2 Tertiary-care hospitals and 2 GI centers	495; 248 SMS 247 Control	SMS 47.5±13.6 Control 47.2±14.8	SMS	Age >18, scheduled for colonoscopy	No indication Morning colonoscopy
					126 (51)		
					Control		
					116 (47)		

GI = gastrointestinal; SMS = short message service; AV = audio-visual video

Characteristics of the mHealth interventions are presented in Table 2. MHealth interventions included smartphone apps [34, 36], SMS text messages [33, 35, 37, 38], smartphone camera app [31], smartphone video clips [29, 30], and a social media app (WeChat) [32, 38]. Operating platforms were Android only, iOS only, or both. All but four studies [30–32, 34] sent reminders to users from one to four days before the colonoscopy appointment as well as on the day of the colonoscopy appointment. The methods of sending reminders included time-alerts, push notifications or text messages. Only Kang et al provided two-way communication using WeChat that allowed patients to ask and receive answers to their questions about the bowel preparation [32]. Jung et al provided a smartphone camera app that analyzed stool images to determine adequacy of the bowel preparation [31]. All mHealth interventions provided laxative and dietary instructions that were heterogeneous in content. Participants in the intervention groups also received the control treatment with the exception of those in the Lorenzo-Zuniga et al study [34].

**Table 2:** Characteristics of the mHealth technologies to support outpatient colonoscopy preparation.

Author	mHealth technology;	Intervention	Control	Laxative
	reminders			
**Sharara et al., 2017 [36]**	Smartphone app;	App contained instructions on diet and laxative regimens, and provided examples and photographs of meals and clear fluids	Written instructions on diet and laxative regimens	Split-dose picosulfate/magnesium citrate
	daily reminders beginning 3 days before and on day of colonoscopy			
**Lee et al., 2015 [33]**	SMS;	Endoscopy nurse used SMS or telephone to provide instructions on diet and laxative regimens	Written instructions on diet and laxative regimens	Split-dose
	telephone or SMS reminders sent 2 days before colonoscopy			2L PEG + ascorbic acid
**Back et al., 2017 [29]**	AV;	3 videos that contained instructions on diet and laxative regimens	Written and verbal instructions on diet and laxative regimens	4 L PEG and low-volume preparation agents including 2 L of PEG/Ascorbic acid or sodium picosulfate with magnesium citrate
	reminders sent 3 days before and on day of colonoscopy			
**Lorenzo-Zuniga et al., 2015 [34]**	Smartphone app;	App provided explanation of colonoscopy, examples of low-fiber diet, pictures of preparation quality; showed video on preparation of the laxative, and a checklist to confirm all steps	Written instructions and visual aids explaining colonoscopy and laxative regimen	Split-dose
	no reminders			2 L PEG+ ascorbic acid
**Kang et al., 2016 [32]**	WeChat;	WeChat provided instructions on diet and laxative regimens and allowed two-way conversation for patients to ask questions and for one investigator to answer questions.	Written and verbal instructions on diet and laxative regimens	Split-dose
	no reminders			PEG
**Jung et al., 2017 [31]**	Smartphone camera app;	App contained instructions on diet and laxative regimens User captures image of feces using app. App automatically rates the bowel preparation quality from the stool status. On the morning of colonoscopy, bowel preparation status checked at every defecation using the app. If “Pass”, they stop taking the solution. If “Fail”, take 150 mL of the solution every 10 min	Written and verbal instructions on diet and laxative regimens	Split dose
	no reminders			4L PEG
**Park et al., 2014 [35]**	SMS;	SMS contained instructions on diet and laxative regimens	Written instructions on diet and laxative regimens	Split dose
	reminders sent day before and 6 hours before colonoscopy			PEG (2L day before, 2L day of)
**Wang et al., 2019 [38]**	WeChat and SMS; reminders sent 2 days before colonoscopy	WeChat contained instructions on diet and laxative regimens. WeChat sent reminder on the appointment and dietary and laxative regimens;	Written instructions on diet and laxative regimens	Split-dose
		SMS group received nurse-provided education		PEG
**Jeon et al., 2019 [30]**	AV;	2 videos contained instructions on diet and laxative regimens and side effects plus nurse-provided explanations	Written and verbal instructions on diet and laxative regimens and side effects	Split-dose
	no reminders			PEG
**Walter et al., 2019 [37]**	SMS;	15 SMS messages contained instructions on diet and laxative regimens	Written and verbal instructions on diet and laxative regimens	Split-dose
	reminders sent 4 days before colonoscopy			2L PEG

SMS = short message service; AV = audio-visual video

PEG = polyethylene glycol

### Risk of bias

**Fig 2:**
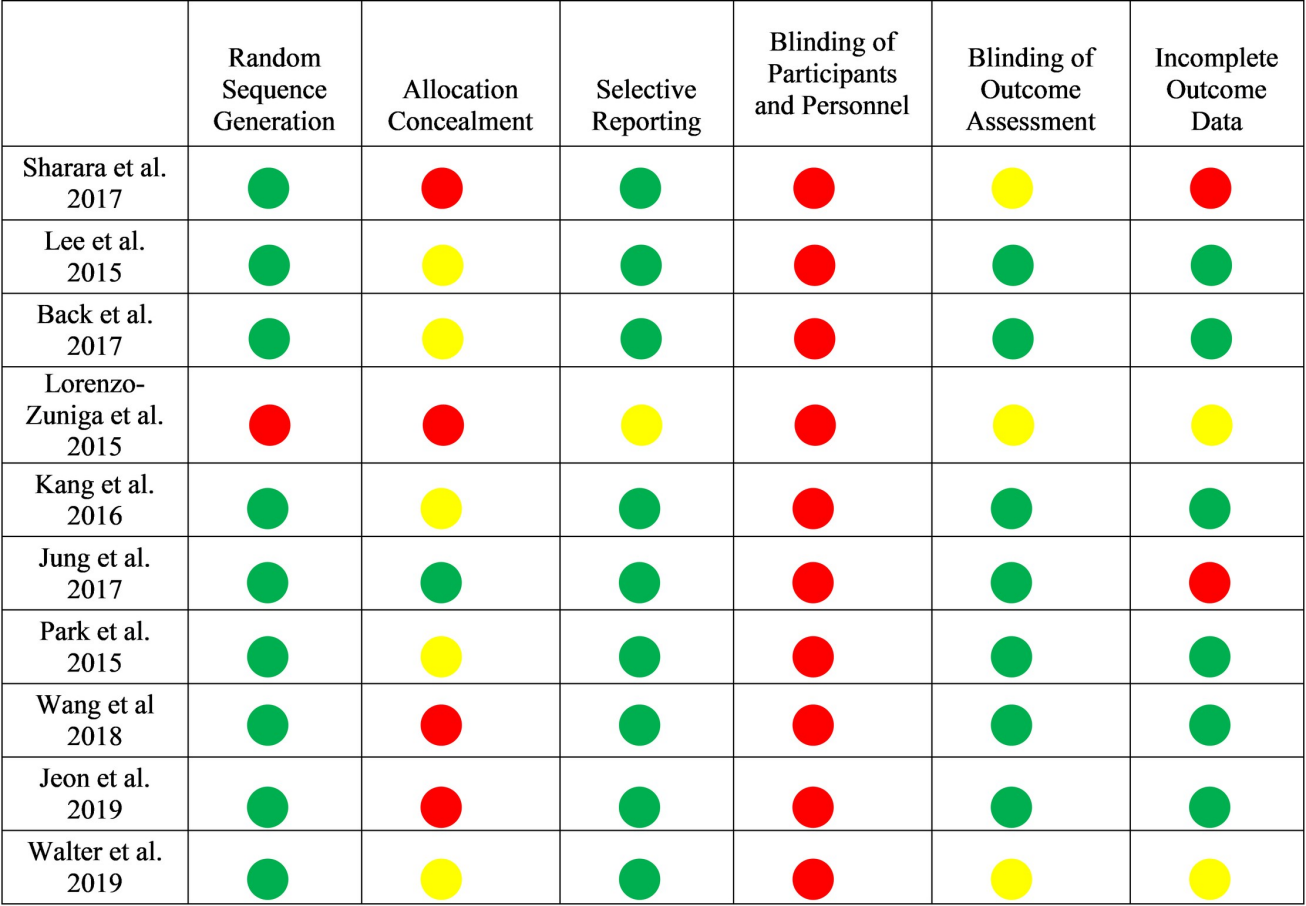
Cochrane risk of bias scores across studies (N = 10).

**Fig 3:**
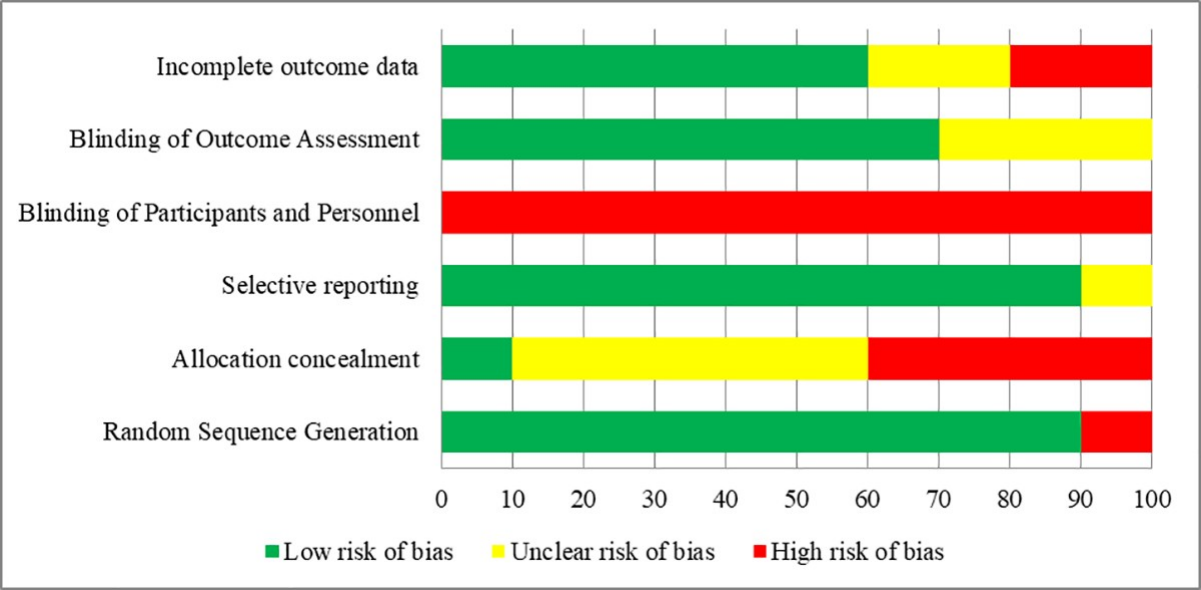
Risk of bias assessment graph.

Assessment of methodological quality showed that all RCTs were at high risk of bias (Figs 2 and 3). None of the trials blinded participants, and in all but one [31] allocation concealment was either unclear or not documented. All studies randomized patients using an acceptable method of randomization, except the Lorenzo-Zuniga et al study, which randomized patients according to the type of smartphone owned [34]. The Sharara et al study was at high risk of bias due to incomplete outcome data, unclear blinding of outcome assessment, and failure to conceal allocation [36]. Four studies [29, 32, 33, 35] had fewer high-risk of bias domains compared to others.

### Outcomes

Table 3 presents a summary of the outcomes assessed in the studies in this systematic review. Outcomes included bowel cleanliness quality, user satisfaction, colonoscopy quality indicators (e.g. cecal intubation time, withdrawal time, adenoma detection rate), adherence to dietary instructions and rates of cancellations and no-shows.

**Table 3:** Summary of outcomes assessed in the trials of mHealth technologies to support outpatient colonoscopy preparation.

Author, Year	Bowel preparation quality scores	Patient satisfaction	Cecal intubation time (minutes)	Withdrawal time (minutes)	Adenoma detection rate	Adherence to diet	Cancellations and no-shows
	mean±SD	aspect; scale	mean±SD	mean±SD	%	%	N
**Sharara et al., 2017 [36]**	OBPS App 6.40±1.95 Control 6.43±1.84 (P = 0.93)	Satisfaction with education method;	Not measured	Not measured	Not measured	Full compliance to diet:	Not measured
	% Adequate bowel preparation	0–10 VAS App 8.7±1.7 Control 8.9±1.1 (no comparison)				App 90.0	
	App 77.2					Control 82.5 (NS)	
	Control 82.5						
	(P = 0.68)						
**Lee et al., 2015 [33]**	BBPS SMS 6.8±1.3 Control 6.3±1.4 (P = 0.03)	Satisfaction with bowel preparation;	SMS 3.5±3.5	SMS 9.8±10.9 Control 9.1±7.6	SMS 21.4 Control 20.4	>70% compliance to diet: SMS 70.9 Telephone 69.8	Cancellations:
	Telephone 7.1±1.2	(5-item scale)	Control 3.4±3.1			Controls 54.7 (P = 0.024)	SMS 12
	Control 6.3±1.4 (P<0.001)	% high/very high:					Telephone 9
		SMS 32.3				>70% compliance to water ingestion:	Control 10
	% Adequate bowel preparation	Telephone 37.3				SMS 63.8 Telephone 50.8	
	SMS 93.7	Control 20.5				Control 44.8 (P = 0.022)	
	Telephone 98.4	(P>0.12)					
	Control 86.1						
	(P<0.001)	% Willing to repeat bowel preparation; Telephone 92.1					
		SMS 89.0 Control 81.8					
		(P = 0.034)					

		SMS vs Control: (P = 0.196)					
**Back et al., 2017 [29]**	BBPS AV 7.53±1.38 Control 6.29±1.83 (P<0.001)	Satisfaction with education method;	Not measured	Not measured	Not measured	Compliance to diet 3 days before colonoscopy	Cancellations:
		0–10 VAS AV 9.16±1.09 Control 7.90±1.94 (P<0.001)				(total score 6):	AV 9
	% Adequate bowel preparation					AV 5.63±0.93 Control 5.06±1.24 (P<0.001)	Control 16
	AV 96.5						
	Control 73.6						
	(P<0.001)						
**Lorenzo-Zuniga et al., 2015 [34]**	HCS App 17.05±3.23 Control 16.52±3.10 (P = 0.19)	Satisfaction with bowel preparation;	Not measured	Not measured	Not measured	Not measured	Not measured
		0–10 NRS SPA 8.7±1.6 Control 6.9±2.7 (P<0.001)					
	% Adequate bowel preparation						
	SPA 100	% Willing to repeat bowel preparation;					
	Control 96.1	SPA 88.9 Control 76.3 (P = 0.007)					
	(P = 0.04)						
**Kang et al., 2016 [32]**	OBPS WeChat 3.6±1.7 Control 4.5±1.8 (P<0.001)	% Willing to repeat bowel preparation; WeChat 91.8 Control 81.0 (P<0.001)	WeChat 7.2±4.6 Control 9.1±4.8 (P = 0.002)	WeChat 7.2±2.2	WeChat 18.6 Control 12.0	Incomplete compliance to diet and laxative: WeChat 12.2 Control 30.1 (P<0.001)	Cancellations:
				Control 7.4±2.1	(P = 0.01)		WeChat 34
	% Adequate bowel preparation			(P = 0.273)			Control 31
	SMS 82.2						
	Control 69.5						
	(P<0.001)						
**Jung et al., 2017 [31]**	OBPS App 2.53±1.26 No-app 2.79±2.06 (P = 0.95)	Acceptability of app;	App 6.88±5.33	App 9.23±5.37	App 21.1 Non-App 29.1 (P = 0.549)	Not measured	No-shows: App 4 Non-App 6
		5-point scale;	Non-App 9.64±4.88 (P = 0.013)	Non-App 9.66±4.88 (P = 0.599)			
	% Adequate bowel preparation	App 4.37±0.90					
	App 94.7						
	Control 79.2						
	(P = 0.15)						
**Park et al., 2014 [35]**	OBPS SMS (median 3, range 0–12) No-SMS (median 5, range 1–9) (P<0.001)	Not measured	Not measured	SMS 6.33±0.79	SMS 30.9 No-SMS 31.1 (P = 0.968)	Compliance to diet:	No-shows:
				No-SMS 6.18±0.66 (P = 0.119)		SMS 88.2	SMS 4 No-SMS 5
	% Adequate bowel preparation					No-SMS 80.0 (P = 0.06)	
	SMS 79.4						
	Control 57.8						
	(P<0.001)						
**Wang et al., 2019 [38]**	BBPS WeChat 6.81 SMS 6.44 Control 5.78	Satisfaction with bowel preparation;	WeChat 7.7±4.4 SMS 8.7±5.4 Control 9.9±5.8 (P = 0.004)	WeChat 6.0±2.5	WeChat 22.0 SMS 18.0 Control 15.2 (P = 0.37)	Compliance to diet:	Cancellations:
		% good/very good WeChat 78.1 SMS 79.1 Control 62.2		SMS 6.2±2.8 Control 7.5±2.9 (P<0.001)		WeChat 88.3	WeChat 3
	WeChat vs SMS (P = 0.007)		WeChat vs Control			SMS 82.9	SMS 2
		WeChat vs Control	(P = 0.001)	WeChat vs Control		Control 70.1 (P = 0.001)	Control 3
	WeChat vs control (P = <0.001)	(P = 0.012)		(P <0.001)			
			SMS vs Control			WeChat vs Control (P<0.001)	
	SMS vs control (P = <0.001)	SMS vs Control (P = 0.009)	(P = 0.087)	SMS vs Control			
				(P < .001)		WeChat vs SMS (P = 0.015)	
	% Adequate bowel preparation	% Willing to repeat bowel preparation;					
	WeChat 89.8	WeChat 89.8					
	Control 66.4	SMS 89.9 Control 92.9 (P = 0.62)					
	(P<0.001)						
**Jeon et al., 2019 [30]**	OBPS AV 5.47±1.74 Control 5.97±1.78 (P = 0.02)	Not measured	AV 2.7±1.6 Control 2.5±1.3 (P = 0.35)	AV 10.2±2.3 Control 10.9±2.2 (P = 0.007)	AV 22.9 Control 34.8	Compliance to food and laxative:	Cancellations;
					(P = 0.028)	AV 97.1	AV 13 Control 12
	% Adequate bowel preparation					Control 91.5 (P = 0.07)	
	AV 50.7						
	Control 42.6						
	(P = 0.17)						
**Walter et al., 2019 [37]**	BBPS SMS 7.4±0.1 Control 6.5±0.1 (P<0.0001)	Burden of preparation;	Not measured	SMS 7.8±0.07 Control 7.7±0.07 (P = 0.19)	SMS 28.6 Control 33.6 (P = 0.52)	Not measured	Cancellations:
		1–10 NRS SMS 5.2±0.1 Control 5.8 ± 0.1 (P = 0.0042)					SMS 2
	% Adequate bowel preparation						Control 3
	SMS 91						
	Control 81						
	(P = .0013)						

OBPS = Ottawa Bowel Preparation Scale; BBPS = Boston Bowel Preparation Scale; HCS Harefield Cleansing Scale

SMS = short message service; AV = audio-visual video

VAS = visual analog scale; NRS = numerical rating scale

#### Bowel cleanliness quality

**Fig 4:**
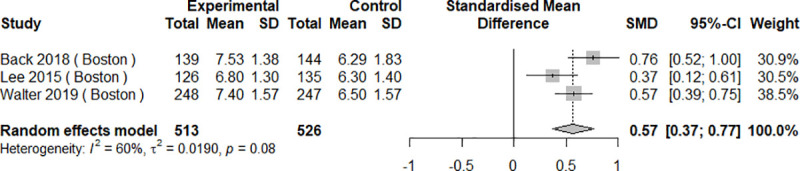
Results of the meta-analysis for bowel preparation quality: Boston Bowel Preparation Scale (BBPS).

**Fig 5:**
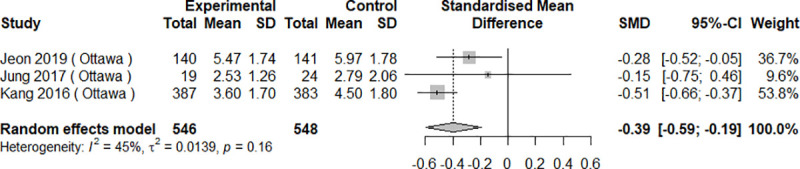
Results of the meta-analysis for bowel preparation quality: Ottawa Bowel Preparation Scale (OBPS).

All ten studies compared bowel cleanliness scores between study groups. Bowel preparation quality measures were heterogeneous and included the BBPS [29, 33, 37, 38], the OBPS [30–32, 35, 36], and the Harefield Cleansing Scale [34]. Sharara et al used three scales, namely the Chicago and Aronchick Scales and the OBPS [36]. In seven studies, mHealth interventions were statistically significantly better compared to control groups [29, 30, 32, 33, 35, 37, 38], while three studies showed no differences between groups [31, 34, 36]. In meta-analysis, mHealth interventions were associated with significantly better bowel cleanliness scores on both BBPS [SMD 0.57, 95%CI 0.37–0.77, I^2^ = 60%, p = 0.08] and OBPS [SMD -0.39, 95%CI -0.59–0.19, I^2^ = 45%, p = 0.16] (Figs 4 and 5). Two studies with insufficient data [35, 38] were excluded from these analyses.

Binary measures of adequate bowel preparation were also compared between study groups. Adequate bowel preparation was defined as scores of ≥ 6 [29, 37, 38] or ≥5 [33] on the BBPS, or <6 [30, 32, 35] or <5 on the OBPS [31]. No cut-offs were reported for the Harefield Cleansing Scale [34] and the Aronchick Scale [36]. In seven studies, statistically significantly higher rates of adequate bowel preparation were reported in the intervention groups compared to the control groups [29, 32–35, 37, 38] while in three studies there were no difference between groups [30, 31, 36]. Rates of adequate bowel preparation were statistically significantly higher in the intervention compared to control groups in three of five studies conducted in South Korea [29, 33, 35], as well as in all four studies with fewer high-risk of bias domains [29, 32, 33, 35].

#### User satisfaction

**Fig 6:**
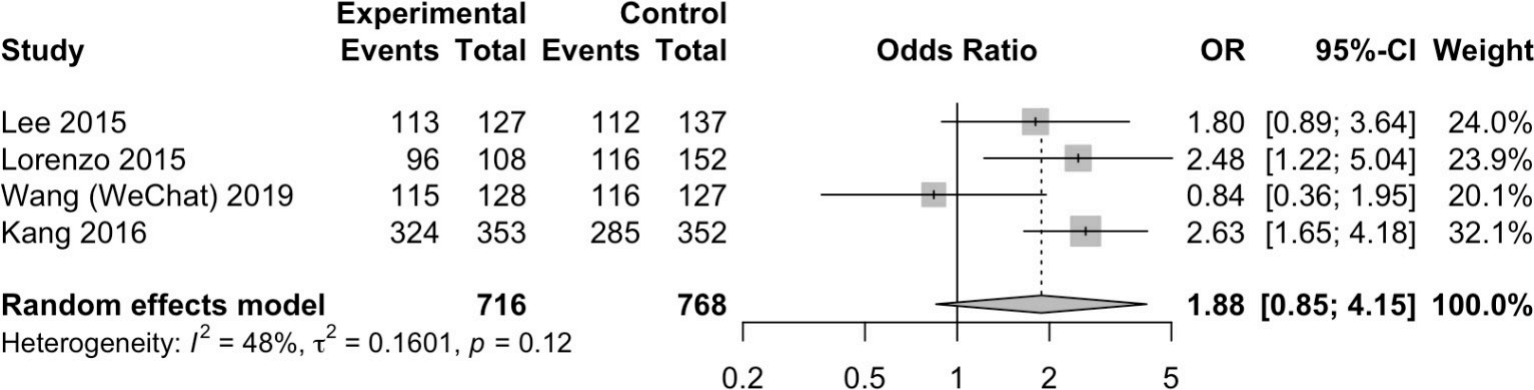
Results of the meta-analysis for willingness to repeat the colonoscopy using the same regimen.

Eight studies assessed various aspects of user satisfaction [29, 31–34, 36–38] using different response scales. The most commonly assessed aspect was willingness to repeat the colonoscopy using the same regimen [32–34, 38], followed by satisfaction with the bowel preparation [33, 34, 38], satisfaction with the method of patient education [29, 36], burden of preparation [37] and acceptability of the app (assessed in the app group only) [31]. The response scales included numerical rating scales (NRS, 1–10 or 0–10) [34, 37], visual analogue scales (VAS, 0–10) [29, 36], a 5-point scale that ranged from very high to very low [33], a 5-point scale that ranged from unacceptable to very acceptable [31], and a 4-point scale that ranged from very good to low [38]. In the studies that compared groups, satisfaction was statistically significantly higher in the intervention groups than in the control groups [29, 32, 34, 37, 38]. In meta-analysis, mHealth interventions were not associated with willingness to repeat the colonoscopy using the same regimen compared to controls [OR 1.88, 95%CI 0.85–4.15, I^2^ = 48%, p = 0.12] (Fig 6).

#### Colonoscopy quality indicators

Seven studies reported the effect of mHealth interventions on colonoscopy quality indicators [30–33, 35, 37, 38]. Although the findings were inconsistent across studies, there were some statistically significant differences between intervention and control groups. Cecal intubation time was lower in the intervention groups in three of four studies [31, 32, 38], withdrawal time was lower in two of five studies [30, 38] and adenoma detection rates, defined as the percentage of individuals with one or more adenomas detected were higher in one study [32], lower in another [30], and similar in four studies [31, 35, 37, 38].

#### Adherence to diet

Seven studies evaluated the effect of mHealth interventions on patient adherence to dietary instruction [29, 30, 32, 33, 35, 36, 38]. Heterogeneous measures of dietary adherence were assessed including rates of complete adherence to diet [35, 36, 38], >70% compliance to diet [33], incomplete compliance with instructions [32], and adherence to diet and laxative [30, 32]. One study assessed compliance to diet restrictions to 6 food types in the 3 days before colonoscopy (total score 6) [29] (Table 2). All intervention groups reported better dietary adherence compared to control groups.

#### Rates of cancellations and no-shows

**Fig 7:**
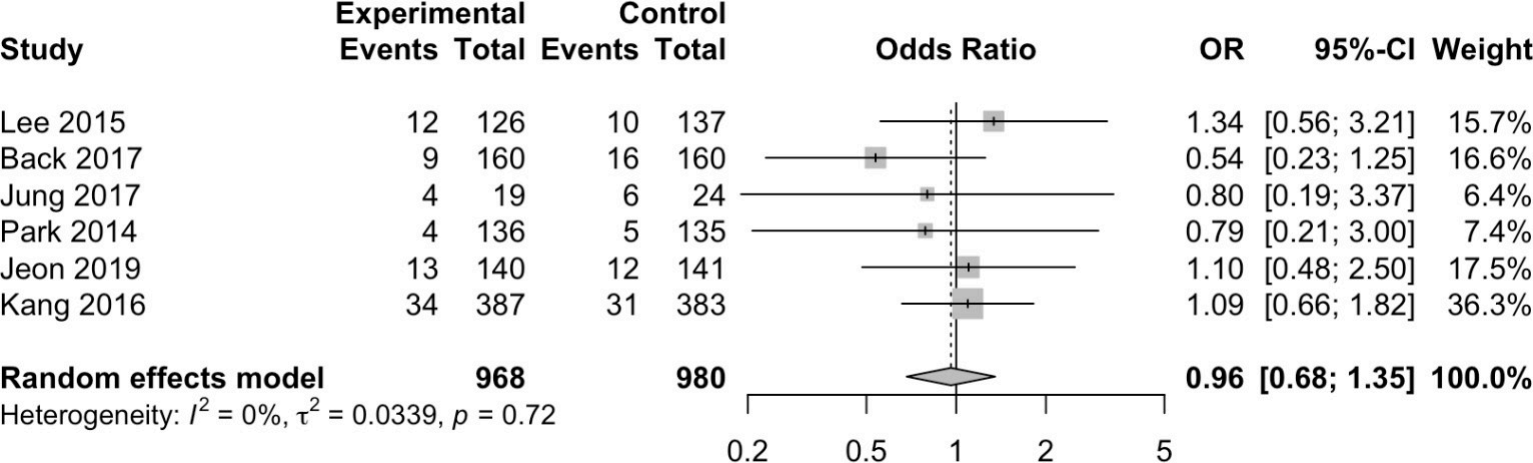
Results of the meta-analysis for cancellation and no-show rates (%).

Eight studies provided information on rates of cancellations/no-shows [29–33, 35, 37, 38]. Of these, six provided the data according to study group but did not assess statistical significance [29–33, 35]. Findings were inconsistent, with three studies showing lower rates in the intervention group compared to controls [29, 31, 35] and three studies showing the reverse [30, 32, 33]. In meta-analysis, mHealth interventions were not significantly associated with the rate of cancellations/no-shows [OR 0.96, 95%CI 0.68–1.35, I^2^ = 0%] (Fig 7).

#### Publication bias

The funnel plot revealed publication bias with respect to adequate bowel preparation (S1 Fig).

## Discussion

We reviewed ten RCTs that examined the effect of mHealth interventions on patient and clinical outcomes in the setting of outpatient colonoscopy. The effect of the mHealth interventions was examined through five outcomes, namely bowel cleanliness quality, user satisfaction, colonoscopy quality indicators, adherence to diet and appointment cancellation and no-show rates. Three studies addressed all five outcomes [32, 33, 38]. Providing patient education using mHealth technologies shows promise as a way to improve the quality of the bowel preparation, as suggested by the analyses of bowel cleanliness scores and the studies with fewer high-risk of bias domains. However, all studies to date are of low methodological quality. There were no associations between mHealth interventions and rates of willingness to repeat the colonoscopy using the same regimen and cancellations/no-shows. The qualitative analyses on adherence to diet and user satisfaction showed these were better in the mHealth intervention groups compared to control groups, but pooled estimates were not generated due to the lack of uniform measures. Finally, mHealth interventions were inconsistently related to colonoscopy quality indicators, possibly because studies were not sufficiently powered to detect group differences.

The content and features of the mHealth interventions examined in this review were heterogeneous. All interventions contained laxative instructions and addressed dietary restrictions, while none addressed the medication restrictions required for bowel preparation. One mHealth intervention study on endoscopy procedures sent general medication instructions that were not tailored to individual patient profiles [39], but this study was not included in the present systematic review due to the reporting of aggregate data for a combination of colonoscopy and gastroscopy procedures in which the majority of procedures was gastroscopies [39]. Nevertheless, mHealth interventions may be effective at improving medication adherence as suggested in a meta-analysis of RCTs in patients with various chronic diseases [40]. An important feature of the mHealth interventions is the reminders that were sent to patients prior to their colonoscopy appointments [29, 33, 35–38]. Two-way communication between a patient and an investigator was an intervention feature that was utilized by 11% of participants in the Kang et al study [32]. The need to comply with jurisdictional privacy legislation to protect patient personal information and be resistant to cyber crime [41] may have discouraged other investigators from incorporating this feature into their interventions.

This systematic review highlights several limitations in the published literature on mHealth technologies supporting colonoscopy preparation. Studies were heterogeneous in populations, inclusion and exclusion criteria, measurement scales, mHealth interventions, and outcome definitions, and some studies failed to use the accepted scale cut-offs for adequate bowel preparation. Another limitation was the low-quality evidence owing to nonblinding of participants and unconcealed allocation. In fact, one trial was a quasi-experimental study where randomization was based on iPhone or Android ownership [34]. The low-quality data analysis and reporting of results are also limitations as all studies reported p-values rather than confidence intervals, and some did not report standard deviations when reporting means. Lastly, although e-health technology developers seeking to improve patient outcomes recommend aligning the capabilities of the technology with end-user needs and preferences [42, 43], none of the RCTs included end-user input in designing the intervention.

Outcomes in the trials reviewed in this study are of interest to both endoscopists who want to provide and patients who want to receive a quality colonoscopy, respectively. Future studies might determine whether mHealth interventions reduce patient pre-procedural anxiety [44] and increase user satisfaction with colonoscopy [45] using validated measures to enhance comparisons across studies and performance of meta-analyses. Future studies might also determine whether aligning end-user needs and preferences with the functionalities of the new technology results in improved patient and clinical outcomes, as well as ensure sufficient statistical power to detect group differences in the colonoscopy quality indicators.

Our findings suggest that patients are more likely to successfully perform the bowel preparation and have greater satisfaction using mHealth technologies compared to written-, verbal- or computer-based instructions. In fact, patients say that the ability of mobile devices to tailor messages to individuals makes them preferable to other forms of education such as brochures, information sessions led by health care professionals or computer-based instructions [46]. MHealth technologies may be particularly economical and useful in open access colonoscopy settings that allow patients to go directly to colonoscopy without a prior visit to an endoscopist, by reducing the need to repeat the colonoscopy due to poor quality bowel cleanliness that, in turn, reduces health care costs and risks to patients.

## Conclusions

In conclusion, mHealth technologies show promise as a way to improve bowel cleanliness in outpatients undergoing colonoscopy. However, they were not associated with colonoscopy quality indicators, willingness to repeat the colonoscopy using the same regimen or rates of cancellation/no-shows. Dietary adherence and user satisfaction were higher in the mHealth intervention groups compared to controls, and the use of validated measures will enhance comparisons across studies and performance of meta-analyses. Given the low methodological quality of the trials conducted to date, high-quality research is required to understand the effectiveness of mHealth technologies on colonoscopy outcomes.

## Supporting information

S1 Checklist PRISMA 2009 checklist. (DOC)

S1 Fig Funnel plot for adequate bowel preparation. (TIF)

S1 Table Literature search strategy. (DOCX)

S2 Table Search strategy for November 2019 update. (DOCX)
