# Supplementary figures and images for: Mobile health technologies supporting colonoscopy preparation: A systematic review and meta-analysis of randomized controlled trials
Source: PLoS One. 2021 Mar 18;16(3):e0248679. doi: 10.1371/journal.pone.0248679 (PMC7971694; doi:10.1371/journal.pone.0248679)

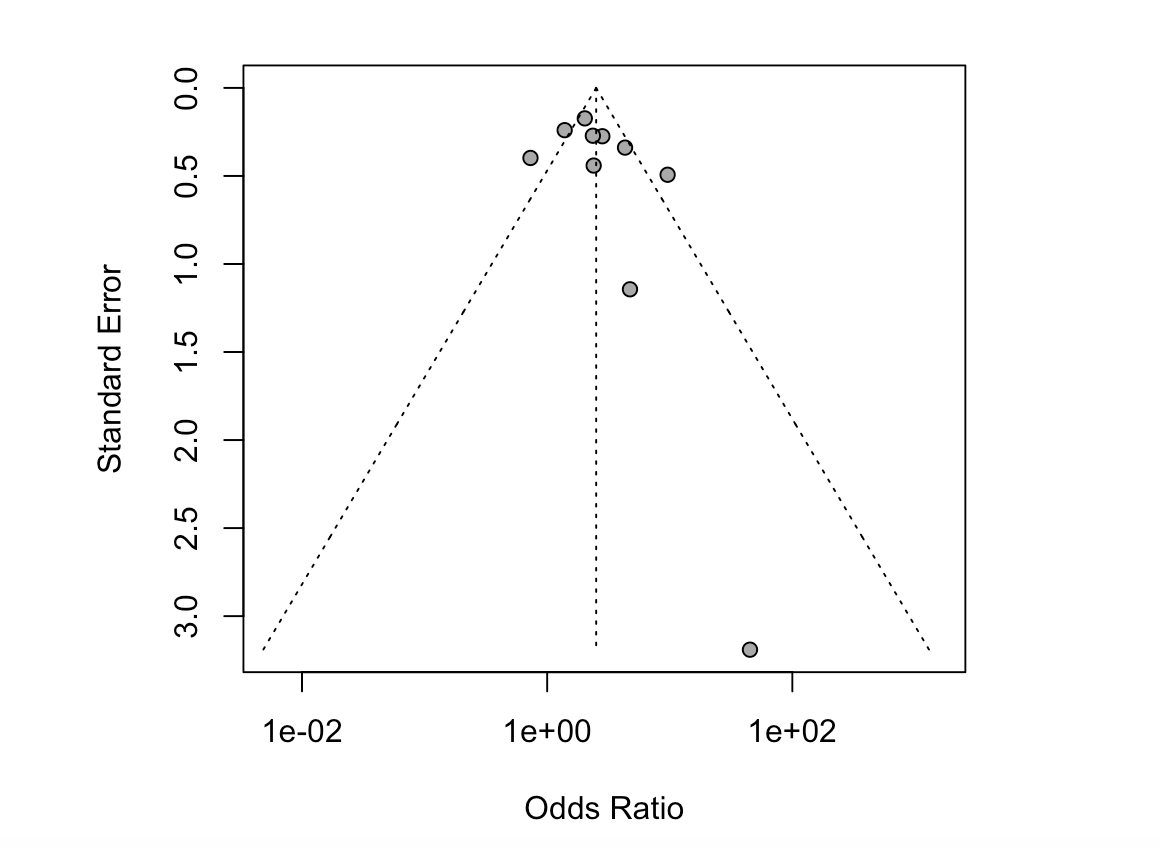

Supplement: S1 Fig — (TIF) [file pone.0248679.s002.tif]
