# Supplementary material for: Mobile health technologies supporting colonoscopy preparation: A systematic review and meta-analysis of randomized controlled trials
Source: PLoS One. 2021 Mar 18;16(3):e0248679. doi: 10.1371/journal.pone.0248679 (PMC7971694; doi:10.1371/journal.pone.0248679)
Supplement: S1 Table — (DOCX) [file pone.0248679.s003.docx]

S1 Table. Literature search strategy

|  |  | Medline | | Embase |  | CINAHL |  | CENTRAL |  | clinicalTrials.g ov |
| --- | --- | --- | --- | --- | --- | --- | --- | --- | --- | --- |
|  |  | Headings | Keyword | Headings | Keyword | Headings | Keyword | Headings | Keyword | Keyword - expert searching |
|  | Colonoscop  y | exp  COLONOSC  OPY/ | colonoscop* OR endoscop* OR bowel  OR bowels  OR colon | exp endoscopy/ or exp colonoscopy/ | colonoscop* OR endoscop* OR bowel  OR bowels  OR colon | (MH  "Colonoscop y+") | colonoscop* OR endoscop* OR bowel  OR bowels  OR colon | MeSH  descriptor: [Colonoscop y] explode all trees | colonoscop* OR endoscop* OR bowel  OR bowels  OR colon | colonoscopy OR endoscopy OR bowel OR bowels OR colon |
| Number  of records |  |  |  |  |  |  |  |  |  |  |
|  | Bowel preparation | exp  Cathartics/ | (bowel adj2 (preparation or prep or preparing or prepared or prepares OR cleans or cleaning or cleaned or cleansing OR lavage OR cathartic* OR purgative*)) OR (colon adj2  (preparation or prep or preparing or prepared or prepares OR | exp laxative/  OR exp  Intestine preparation/ OR exp colon lavage/ | (bowel adj2 (preparation or prep or preparing or prepared or prepares OR cleans or cleaning or cleaned or cleansing OR lavage OR cathartic* OR purgative*)) OR (colon adj2  (preparation or prep or preparing or prepared or prepares OR | (MH "Bowel  Preparation") | (bowel N2 (preparation or prep or preparing or prepared or prepares OR cleans or cleaning or cleaned or cleansing OR lavage OR cathartic* OR purgative*)) OR (colon N2 (preparation or prep or preparing or prepared or prepares OR cleans or | MeSH  descriptor: [Cathartics] explode all trees | (bowel adj2 (preparation or prep or preparing or prepared or prepares OR cleans or cleaning or cleaned or cleansing OR lavage OR cathartic* OR purgative*)) OR (colon adj2  (preparation or prep or preparing or prepared or prepares OR | ("bowel preparation" OR "bowel prep" OR "bowel preparing" OR "bowel prepared" OR "bowel prepares" OR "bowel cleaning" OR "bowel cleans" OR "bowel cleaned" OR "bowel cleansing" OR "bowel lavage" OR "bowel cathartic" OR |

|  |  |  | cleans or cleaning or cleaned or cleansing OR lavage OR cathartic* OR purgative*)) OR (intestin* adj2  (preparation or prep or preparing or prepared or prepares OR cleans or cleaning or cleaned or cleansing OR lavage OR cathartic* OR purgative*)) |  | cleans or cleaning or cleaned or cleansing OR lavage OR cathartic* OR purgative*)) OR (intestin* adj2  (preparation or prep or preparing or prepared or prepares OR cleans or cleaning or cleaned or cleansing OR lavage OR cathartic* OR purgative*)) |  | cleaning or cleaned or cleansing OR lavage OR cathartic* OR purgative*)) OR (intestin*  N2  (preparation or prep or preparing or prepared or prepares OR cleans or cleaning or cleaned or cleansing OR lavage OR cathartic* OR purgative*)) |  | cleans or cleaning or cleaned or cleansing OR lavage OR cathartic* OR purgative*)) OR (intestin* adj2  (preparation or prep or preparing or prepared or prepares OR cleans or cleaning or cleaned or cleansing OR lavage OR cathartic* OR purgative*)) | "bowel purgative") OR ("colon preparation" OR "colon prep" OR "colon preparing" OR  "colon prepared" OR "colon prepares" OR "colon cleaning" OR "colon cleans" OR "colon cleaned" OR "colon cleansing" OR "colon lavage" OR "colon cathartic" OR "colon purgative") OR ("intestinal preparation" OR "intestinal prep" OR  "intestinal preparing" OR  "intestinal prepared" OR "intestinal prepares" OR  "intestinal cleaning" OR "intestinal cleans" OR  "intestinal cleaned" OR |
| --- | --- | --- | --- | --- | --- | --- | --- | --- | --- | --- |

|  |  |  |  |  |  |  |  |  |  | "intestinal cleansing" OR  "intestinal lavage" OR "intestinal cathartic" OR "intestinal purgative") OR ("intestine preparation" OR "intestine prep" OR "intestine preparing" OR "intestine prepared" OR "intestine prepares" OR  "intestine cleaning" OR "intestine cleans" OR "intestine cleaned" OR "intestine cleansing" OR "intestine lavage" OR "intestine cathartic" OR "intestine purgative") |
| --- | --- | --- | --- | --- | --- | --- | --- | --- | --- | --- |
| Number  of records |  |  |  |  |  |  |  |  |  |  |
|  | Smartphone | exp Cell  Phone/ OR exp Computers,  Handheld/  OR exp  SMARTPHO  NE/ OR exp  Mobile  Applications/  OR exp  Reminder Systems/ OR exp Text Messaging/ | ((handheld or hand-held) adj1  (computer? or pc?)) OR (tablet adj1 (device? or comput*)) OR (cell phone* or cell-phone* or cellular  phone*) OR (mobile phone*) OR (smart phone* or  smart-phone* or smartphone*) OR Android OR iPhone?  OR iPad? OR (mhealth or m-health or "m health" or "mobile health") OR (ehealth or ehealth or "e health") OR ((cell phone* or cellphone* or cellular phone* or mobile or smart phone* or smartphone* or | exp mobile phone/ OR exp  smartphone/ OR exp mobile application/ OR exp reminder system/ OR exp text messaging/ | ((handheld or hand-held) adj1  (computer? or pc?)) OR (tablet adj1 (device? or comput*)) OR (cell phone* or cell-phone* or cellular  phone*) OR (mobile phone*) OR (smart phone* or  smart-phone* or smartphone*) OR Android OR iPhone?  OR iPad? OR (mhealth or m-health or "m health" or "mobile health") OR (ehealth or ehealth or "e health") OR ((cell phone* or cellphone* or cellular phone* or mobile or smart phone* or smartphone* or | (MH  "Smartphone  +") OR (MH  "Cellular  Phone+") OR  (MH  "Computers,  HandHeld+") OR  (MH "Mobile  Applications"  ) OR (MH  "Text  Messaging")  OR (MH  "Reminder  Systems") | ((handheld or  hand-held)  N1  (computer? or pc?)) OR (tablet N1 (device? or comput*)) OR (cell phone* or cell-phone* or cellular  phone*) OR (mobile phone*) OR (smart phone* or  smart-phone* or smartphone*) OR Android OR iPhone?  OR iPad? OR (mhealth or m-health or "m health" or "mobile health") OR (ehealth or ehealth or "e health") OR ((cell phone* or cellphone* or cellular phone* or mobile or smart phone* or smartphone* or | MeSH  descriptor: [Cell Phone] explode all trees OR  MeSH  descriptor: [Computers, Handheld] explode all trees OR  MeSH descriptor:  [SMARTPH  ONE] OR MeSH  descriptor: [Mobile  Applications  ] OR MeSH descriptor: [Reminder Systems] explode all trees OR  MeSH  descriptor: [Text  Messaging] explode all trees | ((handheld or hand-held) adj1  (computer? or pc?)) OR (tablet adj1 (device? or comput*)) OR (cell phone* or cell-phone* or cellular  phone*) OR (mobile phone*) OR (smart phone* or  smart-phone* or smartphone*) OR Android OR iPhone?  OR iPad? OR (mhealth or m-health or "m health" or "mobile health") OR (ehealth or ehealth or "e health") OR ((cell phone* or cellphone* or cellular phone* or mobile or smart phone* or smartphone* or | (handheld computer OR hand-held computer) OR  (cell phone) OR (mobile phone) OR (smartphone OR smartphone OR smart phone) OR Android OR iPhone OR iPad OR  (mhealth OR m-health OR m health OR mobile health) OR (ehealth OR e-health  OR e health)  OR  (smartphone app OR smart phone app OR smart-phone app) OR (mobile phone app OR mobile app) |
|  |  |  | smartphone* or Android or iPhone? or iPad?) adj2 (app or apps or  application*)) |  | smartphone* or Android or iPhone? or iPad?) adj2 (app or apps or  application*)) |  | smartphone* or Android or iPhone? or iPad?) N2 (app or apps or  application*)) |  | smartphone* or Android or iPhone? or iPad?) adj2 (app or apps or  application*)) |  |
| Number  of records |  |  |  |  |  |  |  |  |  |  |
|  |  |  |  |  |  |  |  |  |  |  |
| Total |  | 1317 |  |  |  |  |  |  |  |  |
|  |  |  |  |  |  |  |  |  |  |  |
| Searches run Friday, May 4th | |  |  |  |  |  |  |  |  |  |
