# Supplementary material for: Mobile health technologies supporting colonoscopy preparation: A systematic review and meta-analysis of randomized controlled trials
Source: PLoS One. 2021 Mar 18;16(3):e0248679. doi: 10.1371/journal.pone.0248679 (PMC7971694; doi:10.1371/journal.pone.0248679)
Supplement: S2 Table — (DOCX) [file pone.0248679.s004.docx]

**S2 Table. Literature search strategy update.**

**Search strategy**

(Colonoscopy OR Bowel prep) AND smartphone

- Terms used from Excel sheet
- Search terms smartphone – revised with Jill suggestions

**Search documentation**

Summary

| Total | 697 |
| --- | --- |
| Duplicates removed | 81 |
| Deduplicated total | 616 |

Databases

| Database | Ovid Medline |
| --- | --- |
| Database time coverage | 1946-present |
| Date searched | 26 November 2019 |
| Number of records before deduplication | 8 |
| Number of records after deduplication | 8 |

| Database | Ovid Embase |
| --- | --- |
| Database time coverage | 1947-22 November 2019 |
| Date searched | 26 November 2019 |
| Number of records before deduplication | 511 |
| Number of records after deduplication | 496 |

| Database | CINAHL |
| --- | --- |
| Database time coverage | 1937-present |
| Date searched | 26 November 2019 |
| Number of records before deduplication | 73 |
| Number of records after deduplication | 47 |

| Database | Cochrane – Cochrane Reviews |
| --- | --- |
| Database time coverage | 1995-present |
| Date searched | 26 November 2019 |
| Number of records before deduplication | 5 |
| Number of records after deduplication | 5 |

| Database | Cochrane - Trials |
| --- | --- |
| Database time coverage | n/a |
| Date searched | 26 November 2019 |
| Number of records before deduplication | 100 |
| Number of records after deduplication | 60 |
